# Immunologically reactive *M. leprae *antigens with relevance to diagnosis and vaccine development

**DOI:** 10.1186/1471-2334-11-26

**Published:** 2011-01-26

**Authors:** Lucas H Sampaio, Mariane MA Stefani, Regiane M Oliveira, Ana LM Sousa, Greg C Ireton, Steven G Reed, Malcolm S Duthie

**Affiliations:** 1 Tropical Pathology and Public Health Institute, Federal University of Goiás, Rua 235 esquina com 1a Avenida, Setor Universitário, sala 335, Goiânia, GO, 74605050, Brazil; 2 Infectious Disease Research Institute, 1124 Columbia St, Suite 400, Seattle, WA 98104, USA

## Abstract

**Background:**

Leprosy is a chronic infectious disease caused by *Mycobacterium leprae *that can manifest a wide variety of immunological and clinical outcomes ranging from potent humoral responses among borderline lepromatous (BL) and lepromatous (LL) patients to strong cellular responses among tuberculoid (TT) and borderline tuberculoid (BT) patients. Until recently, relatively little has been known about the immune responses to individual proteins of *M. leprae *recognized during leprosy.

**Methods:**

The immune reactivity to a panel of 33 *M. leprae *recombinant proteins was evaluated among leprosy patients and controls from a high endemic area for leprosy (Goiania/GO, Central Brazil). Serum IgG responses were measured by ELISA (45 participants/group) and T cell responses (20 participants/group) were evaluated by IFN-gamma production in 24 hours whole blood cultures with antigen (whole blood assay-WBA). Study groups were newly diagnosed, untreated TT/BT and BL/LL leprosy patients classified by Ridley Jopling criteria and household contacts of BL/LL patients (HHC). Control groups were HIV-1 negative pulmonary tuberculosis patients (TB) and healthy individuals from the same endemic area (EC). In silico predictions indicated the level of identity of *M. leprae *proteins with homologues in other mycobacteria and the presence of T cell and B cell epitopes.

**Results:**

Despite the prediction that all proteins would be reactive, 16 of 33 (48%) of the single proteins tested were immunogenic (recognized in WBA or ELISA) and seventeen were non-immunogenic (not recognized in either assay). Among the 16 immunogenic proteins, 9 were considered leprosy specific in WBA inducing cell-mediated IFN-gamma secretion from TT/BT patients and HHC. Three of these proteins were also leprosy specific in serology being recognized by serum IgG from LL/BL patients. Seven of the immunogenic proteins were not leprosy specific.

**Conclusions:**

New *M. leprae *antigens recognized by antibody responses of BL/LL patients and cellular responses of TT/BT leprosy patients were identified. An improved serological diagnostic test for leprosy could be developed by incorporating these IgG-reactive antigens to the current PGL-I based tests. Moreover our data indicate that the WBA is a robust, relatively simple and user friendly format for a T cell based diagnostic test. The field use of these test formats in leprosy endemic countries could contribute to early leprosy diagnosis before the development of deformities and disabilities.

## Background

Leprosy, caused by infection with *Mycobacterium leprae*, is one of the oldest known human infectious diseases and remains an important public health problem for many countries, including Brazil [1]. *M. leprae *infects macrophages and Schwann cells, causing peripheral nerve damage which results in sensory and motor loss that ultimately cause the severe disability that is a hallmark of leprosy [2]. Leprosy actually manifests across a bacteriologic, clinical, immunologic and pathologic spectrum that allows classification into five forms according to the Ridley-Jopling scale. Weak antibody responses and strong cell-mediated immunity (CMI) classically characterize the immune response of tuberculoid (TT) and borderline tuberculoid (BT) patients who have a low bacterial burden. In contrast, strong antibody responses and weak CMI are classically observed in borderline borderline (BB), borderline lepromatous (BL) and lepromatous (LL) cases that have a high bacterial burden and are believed to transmit *M. leprae *infection [3-5].

Seeking to eliminate leprosy by the year 2000, a campaign by the World Health Organization was based on widespread provision and use of multidrug therapy (MDT) to control infection and reduce transmission. This campaign has produced a large decline in global prevalence of leprosy over the last 20 years, but despite this, the new case detection rate is still high in many regions [1]. The diagnosis of leprosy remains based on the appearance of clinically relevant manifestations and treatment has been simplified to incorporate recommended MDT regimen of 6 months for paucibacillary patients (PB; encompassing TT and BT forms) and of 12 months for multibacillary patients (MB; encompassing LL, BL, BB and some BT forms). Unfortunately, the scarcity of early signs or symptoms, as well as the problem that leprosy symptoms may be confused with other diseases, often leads to significant delays in proper diagnosis and appropriate treatment [6]. A further compounding factor is the reduction in the number of clinicians with expertise at identifying leprosy that has occurred alongside the reduction in case numbers [7]. Early leprosy diagnosis, to promote even earlier treatment, is regarded as critical to provide further reductions in transmission and decrease the number of patients presenting with disabilities [8,9]. To date, simple, rapid tests have been developed to diagnose MB leprosy but tests for PB leprosy are not yet available [10,11].

As with other diseases, rapid and objective diagnosis of leprosy could be achieved by the recognition of disease-specific immune responses [8]. Due to the dichotomous nature of immune responses of leprosy patients, diagnostic tests for all leprosy forms would probably require antigens that are targets of both antibody and cellular responses [8,12]. The identification of T cell-reactive antigens may also reveal proteins that are part of the protective response against leprosy that are worthy of further examination in the context of vaccine development. The selection and production of proteins for immune evaluation has been expedited by recent advances such as the sequencing of the *M. leprae *genome and could be further simplified when coupled with effective in silico epitope predictions [13-18]. Most studies investigating the reactivity of *M. leprae *antigens have described either the antibody or T cell reactivity among leprosy patients but have not typically compared these responses in parallel [4,9,19-26]. The current study was designed to explore the immune reactivity of a panel of 33 *M. leprae *recombinant proteins among leprosy patients and to concomitantly evaluate the utility of computational softwares to predict this reactivity. This study identified several antigens that are targets of the cellular response and some that are targets of the antibody response. Moreover it indicated that the benefits of current computational predictions of immune reactivity were limited.

## Methods

### Study participants

This study was approved by local (Comitê de Ética em Pesquisa Humana e Animal do Hospital das Clínicas da Universidade Federal de Goias) and National Ethics Commission (Comissão Nacional de Ética Pesquisa/CONEP/Brazil protocols #4862 and #12962). Newly diagnosed, previously untreated leprosy patients were recruited between November 2006 and January 2009 at a main outpatient clinic (Centro de Referência em Diagnóstico e Terapêutica) in Goiânia, Goiás State, in Central-West Brazil. Before recruitment all patients were provided complete dermato-neurological evaluations by a dermatologist with expertise in leprosy diagnosis. Participants were included only after signing written informed consent forms. Patients were assigned to leprosy groups based upon their immediate diagnosis, and were subsequently thoroughly characterized according to Ridley-Jopling criteria, taking into account clinical, bacilloscopic and histopathological findings [27]. All patients assigned to the TT/BT group had negative bacilloscopic index (BI), whereas LL/BL cases were BI positive. Tuberculosis patients (TB), healthy household contacts of LL/BL patients (HHC) and healthy endemic controls (EC) from the same endemic region were included as control groups. TB patients were HIV-negative individuals with clinically confirmed pulmonary tuberculosis (*Mycobacterium tuberculosis *sputum-positive) in their final 3 months of chemotherapy. HHC were adults living in the same house as a multibacillary index case for at least 6 months prior to study enrollment and blood collection. EC were healthy individuals recruited among non leprosy patients at a public health center from the same endemic setting. EC had neither tuberculosis nor history of leprosy in their families. All study participants had a BCG scar, consistent with the neonatal BCG vaccination coverage close to 100% in this recruitment setting. All study participants came from Goias State, an endemic region for leprosy (prevalence rate = 6.02 patients/10000 inhabitants). The serology study included 45 participants per group and the T cell study included 20 participants per group and results from these separate studies were combined in this manuscript. The fine composition of each study group is summarized in Table 1.

**Table 1:** Main characteristics of study groups and anti PGL-I serology data.

Group	Sex ratio (m/f)	Mean age (years, range)	BI (mean, range)	PGL-I serology mean OD (range)
PB (29 TT/36 BT)	31/34	33 (18-76)	0 (0)	0,19 (0 - 1,03)
MB (23 BL/42 LL)	37/28	51 (18-100)	3.75 (0.5-6.0)	0,82 (0,08 - 2,76)
HHC	32/33	36 (19-60)	na	0,09 (0 - 0,53)
TB	39/26	38 (20-67)	na	0,07 (0 - 0,30)
EC	32/33	35 (18-58)	na	0,07 (0,01 - 0,30)

PB: paucibacillary leprosy consisting of TT and BT forms; MB: multibacillary leprosy consisting of BL and LL forms; m/f: male/female ratio; BI- bacilloscopic index; Optical density (OD) of IgM antibodies to PGL-I antigen detected by ELISA; na: not applicable.

### Antigens

Thirty-three *M. leprae *recombinant proteins were evaluated for immune reactivity (ML0022, ML0051, ML0098, ML0176, ML0276, ML0393, ML0405, ML0489, ML0491, ML0540, ML0810, ML0811, ML0840, ML1383, ML1556, ML1632, ML1181, ML1481, ML1633, ML1685, ML2028, ML2044, ML2055, ML2203, ML2331, ML2346, ML2358, ML2380, ML2541, ML2603, ML2629, ML2655 and ML2659). Proteins were selected either from previous *M. leprae *cDNA library screening results [28] or on the basis of homology with known secreted *M. tuberculosis *proteins. Cloning and purification from *E. coli *were performed as previously described [4,28]. Endotoxin levels for each protein were <100 EU/mg protein, as measured by Limulus Amebocyte Lysate QCL-1000 assay (Lonza Inc., Basel, Switzerland). Details for selected *M. leprae *recombinant proteins are provided in Table 2.

**Table 2:** Representative *M. leprae *recombinant antigens examined by comparative genomics and actual immune responses.

		**% Amino acid identity and orthologous genes**^**1**^						**Number of Predicted epitopes**^**2**^		**Immunogenicity specificity**^**3**^	
**Gene**	**Predicted Function**	**Size (aa)**	***M. ulcerans***	***M. avium***	***M. bovis ***(BCG)	***M. marinum***	***M. tuberculosis***	**T cell**	**B cell**	**WBA IFNγ**	**Ab**
ML0405	hypothetical protein	394	29 (MUL_5045)	-	62 (BCG_3680c)	57 (MMAR_4515)	62 (Rv3616c)	7	39	+/+	+/+
ML2331	possible secreted protein	256	81 (MUL_4308)	80 (MAV_0385)	82 (BCG_3777)	81 (MMAR_5233)	81 (Rv3717)	4	24	+/+	+/+
ML2055	fibronectin attachment protein	287	65 (MUL_3017)	57 (MAV_2859)	67 (BCG_1896)	65 (MMAR_2737)	67(Rv1860)	6	15	+/+	+/+
ML1685	3-isopropylmalate dehydratase	476	86 (MUL_1968)	88 (MAV_3838)	88 (BCG_3009c)	88 (MMAR_1726)	88 (Rv2988c)	3	25	+/+	-
ML1632	possible hydrolase	511	73 (MUL_1334)	77 (MAV_2243)	75 (BCG_2040c)	74 (MMAR_3295)	75 (Rv2223c)	4	32	+/+	-
ML1556	initiation factor IF-2	924	83 (MUL_2122)	81 (MAV_3693)	83 (BCG_2859c)	82 (MMAR_1894)	83 (Rv2839c)	3	17	+/+	-
ML2044	Possible glycosyl hydrolase (pseudogene)	73	-	-	-	-	-	3	7	+/+	-
ML0840	hypothetical protein	434	-	66 (MAV_2053)	-	-	-	3	22	+/+	-
ML0276	hypothetical protein	147	-	71 (MAV_4774)	78 (BCG_0427)	70 (MMAR_0687)	78 (Rv0390)	5	14	+/+	+/-
ML2541	acyl-CoA synthase (pseudogene)	146	77 (MUL_0004)	78 (MAV_0004)	83 (BCG_0004)	78 (MMAR_0004)	77 (Rv0004)	5	15	+/-	-
ML2203	hypothetical protein	373	76 (MUL_0420)	76 (MAV_0750)	77 (BCG_0863c)	75 (MMAR_4878)	77 (Rv0811c)	6	17	+/-	-
ML2358	probable acyl-CoA synthase	583	77 (MUL_2020)	56 (MAV_1328)	77 (BCG_2952)	77 (MMAR_1777)	76 (Rv2930),	3	15	+/-	-
ML2346	hypothetical protein	301	-	-	-	-	-	5	12	+/-	-
ML2380	possible secreted protein	153	65 (MUL_1413)	66 (MAV_4695)	66 (BCG_0494c)	65 (MMAR_0777)	66 (Rv0455c)	3	15	+/-	-
ML2603	possible lysophospholipase	279	73 (MUL_1077)	77 (MAV_4997)	74 (BCG_0220)	74 (MMAR_0427)	74 (Rv0183)	5	16	+/-	-
ML0022	hypothetical protein	488	71 (MUL_0024)	66 (MAV_0024)	72 (BCG_0050c)	69 (MMAR_0022)	72 (Rv0020)	5	17	+/-	-

^1 ^Blast Search for amino acid identity was performed using BLAST Uniprot (http://www.uniprot.org/). (-) = no homologue found. Locus_tag othologous genes are indicated within parentheses.

^**2 **^Promiscuous T cell epitopes predicted to be recognized by least 26 of 51 different HLA-DR alleles identified by PROPED server (http://www.imtech.res.in/raghava/propred/), and the number of potential B cell epitopes predicted by ABCpred server (http://www.imtech.res.in/raghava/abcpred/ABC_submission.html).

^3^Immu/spec = immunogenicity/specificity; WBA +/+ = immunogenic protein that gives specific cellular response; WBA +/- = immunogenic protein that gives unspecific cellular response; Ab +/+: immunogenic protein that induces specific antibody response; Ab+/-: immunogenic protein that induces unspecific antibody response. (-) = not immunogenic.

### CMI determination by whole blood assay (WBA)

WBA was performed as previously described [4]. Briefly, at the time of initial diagnosis and operational assignment to MB or PB groups, prior to full characterization by Ridley-Jopling, undiluted heparinized venous whole blood (Greiner) was collected. Whole blood was plated into 24-well plates (450 μl/well; Sigma, St. Louis, MO) within 2 hours of collection and incubated with stimulants for 24 hours at 37°C 5% CO_2_. Each assay included stimulation with 10 μg/ml of individual *M. leprae *recombinant protein for experimental evaluations, and with PBS alone, 10 μg/ml *M. leprae *cell sonicate (MLCS; provided by Dr. John Spencer, Colorado State University, Fort Collins, CO under NIH contract N01 AI-25469) or 1 μg/ml PHA (Sigma) as controls (Additional File 1). Approximately 150 μl plasma were collected and stored at -20°C until IFNγ determination. IFNγ concentration was determined by ELISA, according to the manufacturer's instructions (eBioscience kit, San Diego, CA). The IFNγ ELISA employed had a detection limit of 20 pg/ml and an arbitrary cut-off point of 50 pg/ml was used to determine positive responses.

### Antibody ELISA to *M. leprae *recombinant proteins

Serum IgG antibodies were determined by ELISA as previously described [3]. Polysorp 96-well plates (Nunc, Rochester, NY) were coated with 2 μg/ml recombinant protein at 4°C and blocked with PBS/Tween-20 with 1% (wt/vol) BSA. Serum samples diluted 1/200 in 0.1% BSA were added and incubated for 1 hour at RT. Plates were washed and incubated with horseradish peroxidase-conjugated anti-human IgG (Southern Biotech, Birmingham, AL). After washing, reactions were developed with peroxidase color substrate (KPL, Gaithersburg, MD), and quenched by the addition of 1N H_2_SO_4_. The corrected optical density of each well at 450 nm was read using a VERSAmax microplate reader (Molecular Devices, Orleans Drive Sunnyvale, CA). Responses were defined as positive if the median was above the arbitrary cut-off (OD >0.3).

### ELISA to PGL-I antigen

IgM antibodies to *M. leprae *cell wall phenolic glycolipid I (PGL-I) were detected as described [3]. Briefly 200 ng/ml of natural disaccharide with octyl linkage (NDO) conjugated to bovine serum albumin (NDO-BSA; kindly supplied by John Spencer, Colorado State University, under NIH contract N01 AI-25469) was used. Serum samples diluted 1/300 in 0.1% BSA were tested in duplicates. After incubation and washings horseradish peroxidase-conjugated to anti human IgM (Rockland Immunochemicals, Gilbertsville, PA) was added and incubated. After washings peroxidase color substrate (KPL, Gaithersburg, MD) was added and the reaction was quenched by the addition of 1 N H2SO4. The optical density (OD) was read at 450 nm. Positive responses were defined as an OD of 2x the mean OD of healthy endemic controls. Results of IgM anti PGL-I serology in each study group are shown in Table 1.

### Comparative genomics and epitope predictions

The amino acid sequences of the *M. leprae *proteins evaluated in this study were obtained from the Leproma website (available at http://genolist.pasteur.fr/Leproma/) and the SANGER CDS Retrieve Information (available at http://www.sanger.ac.uk/cgi-bin/yeastpub/get_cds?organism=M_leprae) [14,16]. The percentage of amino acid identity of each *M. leprae *protein tested was assessed against homologues in other mycobacteria that were revealed by the comparative protein analyses based on complete proteome databases from other mycobacteria that have the potential to infect humans: *M. tuberculosis*, *Mycobacterium avium*, *Mycobacterium smegmatis*, *Mycobacterium marinum*, *Mycobacterium ulcerans, Mycobacterium bovis *BCG, *Mycobacterium paratuberculosis *and *Mycobacterium microti. *BLAST UniProt (The Universal Protein Resource) database (available at http://www.uniprot.org) was used to compare a given amino acid sequence with sequences of other proteins from the NCBI database indicating sequences with identity above 30% [15,29]. The potential of the selected *M. leprae *proteins to present promiscuous MHC class II T cell epitopes was predicted by PROPRED server (available at http://www.imtech.res.in/raghava/propred/) which uses a panel of 51 different HLA-DR alleles (HLA-DRB1 and HLA-DRB5) [13,30]. The prediction of B cell epitopes containing 16 amino acids in the selected *M. leprae *proteins was attained using the artificial neural network based server ABCpred (available at http://www.imtech.res.in/raghava/abcpred/ABC_submission.html) [17,31].

### Statistical analysis

Exploratory data analysis, including box-plots and scatter plots, medians and interquartile range (IQR) were used to analyze the IFNγ levels and OD of IgG reactivity among different study and control groups. Proteins that demonstrated reactivity above the arbitrary cut off (OD >0.3 for IgG ELISA or IFNγ secretion >50 pg/ml in WBA) were classified as "immunogenic". For T cell recognition *M. leprae*-specificity was defined according to reactivity of proteins among TT/BT leprosy patients and at risk HHC, and lack of reactivity among TB and EC groups. For serology *M. leprae *specific proteins were recognized by IgG antibodies from leprosy patients especially BL/LL categories and by lack of recognition by HHC, EC and TB groups. Statistical significance was assessed by Kruskall-Wallis one way analysis of variance for comparison of multiple groups and Mann-Whitney for comparison between two groups. Results were considered statistically significant when *p*-values < 0.05 were obtained.

## Results

### ML0405, ML2055 and ML2331 proteins elicit leprosy-specific cellular and humoral responses

**Figure 1:**
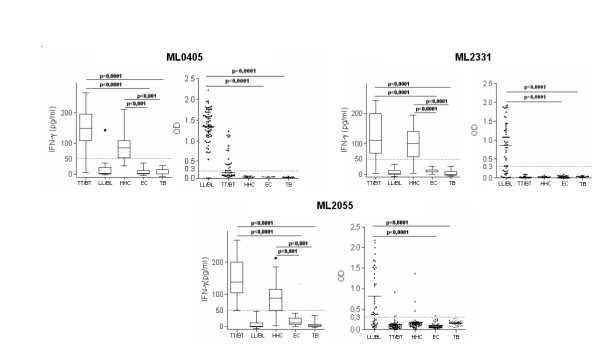
**Identification of leprosy-specific antigens by cellular and antibody responses**. WBA-IFNγ secretion and serum IgG responses were determined for ML0405, ML2331 and ML2055. For WBA blood from leprosy patients and controls were incubated for 24 h with ML0405, ML2331 and ML2055 antigens, and IFNγ secretion determined by ELISA. The dotted lines represent the arbitrary cut-off IFNγ above 50 pg/ml. WBA results are presented as box plots encompassing the 25th and 75th percentiles, and the black line within each box indicating the median value, n = 20 per group. IgG ELISA data are shown in scatter plots of individual values for each individual serum tested, n = 45 per group. The dotted line indicates the calculated cut-off for positive responses (OD >0.3).

Dependent upon their disease presentation, leprosy patients can be characterized as having strong antibody responses (MB; LL/BL) or strong cellular responses (PB; TT/BT) against crude *M. leprae *antigens. It is unclear if these different patient groups respond to the same or different individual antigens. To address this question, we compared the antigen-specific immune response of leprosy patients and controls. When incubated with blood from TT/BT and HHC groups, the ML0405, ML2055 and ML2331 proteins induced strong IFNγ production (Figure 1). The ML0405 and ML2055 antigens each induced secretion of IFNγ greater than 50 pg/ml in 95% (19 of 20) of TT/BT leprosy patients examined. The ML2331 antigen induced response greater than 50 pg/ml IFNγ in 85% (17 of 20) of the TT/BT cases examined. The ML0405 and ML2331 proteins also induced production of IFNγ levels above the 50 pg/ml cut-off among 80% HHC (16 of 20 cases to each antigen), consistent with this group being exposed to *M. leprae*. The ML2055 antigen similarly induced secretion of IFNγ greater than 50 pg/ml in 75% HHC (15 of 20 cases). In contrast, with the exception of one LL/BL patient responding to ML0405 and one EC responding to ML2055, IFNγ responses above 50 pg/ml were not observed in the LL/BL leprosy or control groups (Figure 1).

Although not recognized in terms of IFNγ production within WBA with LL/BL blood, as we previously reported, the ML0405 and ML2331 proteins were well recognized by serum IgG from LL/BL leprosy group (Figure1) [3,28]. The ML2055 antigen demonstrated a strong serum antibody reactivity in the LL/BL patient group (median OD = 1.43; IQR = 1.26) with 58% (26 of 45) sera binding above the positive threshold (OD >0.3; Figure 1). Antibody responses to ML0405, ML2055 and ML2331 within all other groups analyzed (TT/BT, HHC, TB and EC) were weak or absent. Taken together, the data indicate that ML0405, ML2055 and ML2331 are recognized specifically among leprosy patients and contacts, albeit differentially inducing either antibody or cellular responses.

### Antigens that stimulate leprosy-specific CMI without specific antibody responses

**Figure 2:**
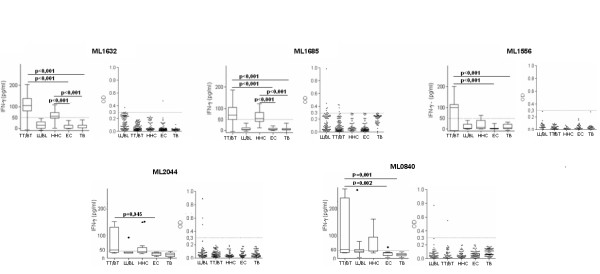
**Proteins that are specifically recognized by cellular responses do not necessarily elicit serum antibodies**. WBA-IFNγ secretion and serum IgG responses were assessed against the ML1632, ML1685, ML1556, ML2044 and ML0840 proteins. WBA results are presented as box plots encompassing the 25th and 75th percentiles, and the black line within each box indicating the median value, n = 20 per group. Antibody results are shown in scatter plots of individual values for each individual serum tested, n = 45 per group. The dotted line indicates the calculated cut-off for positive responses (OD >0.3).

Five proteins were found to elicit cellular responses despite not being detected by serum antibodies. In agreement with our previous findings, antigens ML0840 and ML2044 induced significantly greater IFNγ secretion in WBA using blood from TT/BT leprosy patients than WBA using EC blood (Figure 2) [4]. These proteins did not, however, demonstrate significant antibody reactivity in any of the study groups (Figure 2). Among the TT/BT patients, IFNγ production was induced by incubation with the ML1632 protein in 80% (16 of 20) cases; by the ML1685 and ML1556 proteins in 75% (15 of 20) cases. Antigen-specific IFNγ responses were again induced in the HHC samples, with 65% HHC (13 of 20) responding to the ML1632 and ML1685 proteins, and 25% HHC (5 of 20) responding to the ML1556 protein (Figure 2). Thus, our data identifies five *M. leprae *proteins (ML0840, ML2044, ML1632, ML1685 and ML1556) that, despite stimulating IFNγ production in WBA using blood from the TT/BT and HHC groups, are not detected by serum IgG responses of leprosy patients.

**Figure 3:**
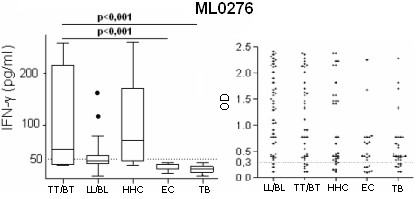
**The ML0276 protein elicits a leprosy-specific cellular response but is detected by serum antibodies from all study groups**. WBA-IFNγ and serum IgG responses against ML0276 are shown as an example of a protein specific for WBA IFNγ but that lacks specificity for serum IgG responses. WBA results are presented as box and whisker plots, with the boxes encompassing the 25th and 75th percentiles, and the black line within each box indicating the median value, n = 20 per group. Antibody results are shown in scatter plots of individual values for each individual serum tested, n = 45 per group. The dotted line indicates the calculated cut-off for positive responses (OD >0.3).

We previously demonstrated that the ML0276 protein induces IFNγ secretion from TT/BT leprosy patient and HHC blood but not from controls [4]. When the ML0276 protein was used in ELISA to detect antibodies, strong serum IgG reactivity was observed in all study groups (Figure 3). Our data thereby indicate that although the cellular response to ML0276 is specific to leprosy groups, the anti-ML0276 antibody response lacks specificity. Medium values of IFNγ produced in WBA and OD of ELISA tests to detect IgG to the immunogenic *M. leprae *recombinant proteins identified in this study, stratified by different study groups are shown in Additional File 2.

### Non-specific cellular responses are observed against many proteins

**Figure 4:**
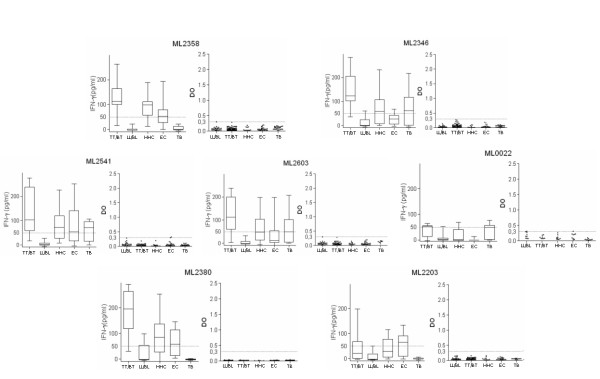
***M. leprae *proteins that lack specificity**. The proteins ML0022, ML2358, ML2346, ML2380, ML2541, ML2603 and ML2203 were immunogenic but not specific. WBA results are presented as box and whisker plots, with the boxes encompassing the 25th and 75th percentiles, and the black line within each box indicating the median value, n = 20 per group. Antibody results are shown in scatter plots of individual values for each individual serum tested, n = 45 per group. The dotted line indicates the calculated cut-off for positive responses (OD >0.3).

In contrast with the leprosy-specific cellular responses of the proteins categorized above, several of the recombinant proteins tested, in addition to the TT/BT leprosy group, stimulated IFNγ release in the TB or EC groups (ML0022, ML2358, ML2346, ML2380, ML2541, ML2603 and ML2203) (Figure 4). Of note, the LL/BL leprosy group did not respond to these proteins. The number of responders to each of these proteins, and the magnitude of the response, were generally also much lower than those observed for the proteins that were specifically recognized by TT/BT leprosy group. Despite the presence of cellular responses, these proteins did not demonstrate IgG reactivity.

### In silico predictions of immune reactivity and specificity are poor

Comparative analyses of amino acid sequences of the *M. leprae *proteins evaluated immunologically were conducted against the predicted proteomes of *M. tuberculosis*, *M. avium*, *M. marinum*, *M. ulcerans*, *M. bovis *BCG (Table 2), *M. smegmatis, M. paratuberculosis *and *M. microti *(Additional File 3). Only the ML2346 protein could be considered *M. leprae *unique, with no homologues found in the other mycobacteria species examined (Table 2). The other *M. leprae *proteins tested herein possessed an ortholog protein in other pathogenic mycobacteria, with homology ranging from 29% to 97% (Table 2 and Additional File 3). This high homology was not, however, associated with a lack of specificity in ex vivo assays. For example, the ML2331 protein possesses 81% homology with the Rv3717 ortholog in *M. tuberculosis*, but this protein was not reactive with TB patient samples and responses against ML2331 could only be demonstrated in leprosy patients (or HHC). Thus, despite possessing significant levels of homology with other mycobacteria species, several *M. leprae *proteins are only recognized in the context of leprosy.

**Figure 5:**
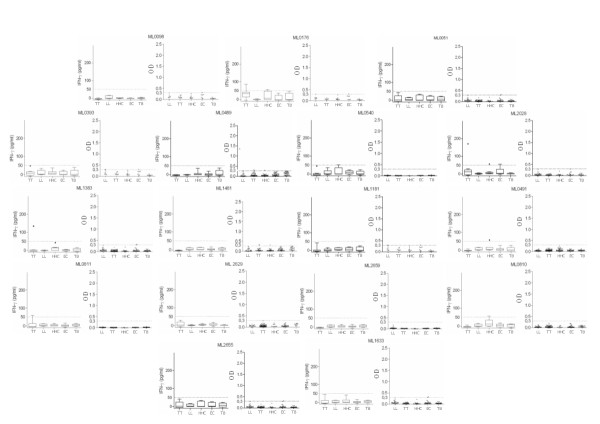
***M. leprae *proteins that are not reactive in WBA or antibody ELISA**. WBA-IFNγ secretion and serum IgG responses were assessed against the proteins ML0051, ML0098, ML2028, ML0176, ML1633, ML0393, ML0489, ML2655, ML0491, ML0540, ML0810, ML1383, ML1481, ML2629, ML2659, ML0811 and ML1181. WBA results are presented as box and whisker plots, with the boxes encompassing the 25th and 75th percentiles, and the black line within each box indicating the median value, n = 20 per group. Antibody results are shown in scatter plots of individual values for each individual serum tested, n = 45 per group. The dotted line indicates the calculated cut-off for positive responses (OD >0.3).

Having found widely discrepant levels of reactivity in our immune assays, we retrospectively compared our results with in silico predictions to determine if computational analyses could have streamlined the in vivo assays. In general, the number of predicted B cell and T cell epitopes was directly proportional to the size of the protein (Table 2). In silico predictions indicated that each of the proteins tested contained up to seven promiscuous T cell epitopes, indicating that all had the potential to be reactive in WBA (Table 2). This prediction clearly contradicts our observation that more than half of the antigens tested (ML0051, ML0098, ML2028, ML0176, ML1633, ML0393, ML0489, ML2655, ML0491, ML0540, ML0810, ML1383, ML1481, ML2629, ML2659, ML0811 and ML1181) neither induced IFNγ secretion nor showed IgG reactivity (Figure 5). The in silico predictions of HLA binding regions within an antigen sequence do not predict interactions with the T cell receptor (TCR). The analyses employed herein were poor to determine the proteins that are targets of the adaptive immune response of leprosy patients.

## Discussion

Early and accurate diagnosis of leprosy and MDT are considered essential to disrupt *M. leprae *transmission and leprosy incidence. Due to the complex and varying immune responses that characterize leprosy spectrum, it is likely that immune diagnosis will only be achieved by antigens that specifically induce cellular and humoral responses [8,19]. Identifying antigens that are targets of the proinflammatory T cell response could also reveal those that are associated with limiting *M. leprae *burden and therefore useful for vaccination. In this study a panel of recombinant *M. leprae *proteins was evaluated for the ability to induce CMI and antibody responses to identify antigens that are specifically reactive in leprosy patients. Although many antigens were identified as cellular targets by their ability to induce IFNγ secretion in WBA of TT/BT leprosy patients and HHC, a minority of the screened proteins induced serum IgG responses in BL/LL patients, such that few proteins induced specific responses across the leprosy spectrum.

In silico analysis predicted the presence of T cell and B cell epitopes within all of the *M. leprae *proteins tested in this study. The most promiscuous T cell epitope predicted was located in ML0405 protein, which was estimated to contain T cell epitopes recognized by 50 of 51 of HLA-DR alleles along with 24 potential B cell epitopes. Consistent with this prediction, the ML0405 protein was recognized by IFNγ release in WBA using TT/BT leprosy patient blood and by serum IgG from LL/BL leprosy patients. However, despite the prediction that all of the evaluated proteins would possess multiple HLA-DR epitopes, more than half (17 of 33; 52%) of the proteins were not recognized by leprosy patients or controls. Our results are in agreement with previous studies that found some of these proteins were not immunogenic [4,9,19-25]. Although we cannot exclude the possibility that the proteins investigated were not recognized because individuals with the appropriate HLA were not recruited, we consider this improbable given the large degree of promiscuity that was predicted. Several other possibilities could explain the lack of reactivity. Although we consider it unlikely given the recognition of positive responses to many proteins, the simplest explanation would be that recombinant expression in E. coli leads to significantly different folding and processing of proteins than occurs during native expression in *M. leprae*. A more likely possibility is that these proteins, although present in the genome of *M. leprae*, may not be translated [16]. Another explanation could be that limited antigen-presentation or T cell suppression may occur during leprosy in order to prevent nerve damage via T cell-mediated killing of *M. leprae*-infected Schwann cells. We are aware that the softwares employed in this study identify and predict HLA binding regions from antigen sequences without predicting interactions with the TCR. Therefore the use of other in silico prediction softwares that also include the probability of the antigen being processed, presented in the context of a certain HLA allele and recognized by TCR could lead to different conlusions.

In leprosy endemic regions the high rates of concomitant exposure to *M. tuberculosis *and to other non-pathogenic environmental mycobacteria could confound the determination of *M. leprae*-specific immune responses. Several of the *M. leprae *antigens tested in this study have homologues in other mycobacteria with greater than 50% identity (Table 1). The routine *M. bovis *BCG vaccination of newborns in leprosy-endemic countries such as Brazil could stimulate responses that could cross-react with *M. leprae*. *M. bovis *BCG showed high homology with immunogenic *M. leprae *proteins (from 62 to 88%) This high homology was not, however, associated with neither cross -reactivity nor with a lack of specificity. For example, the ML2331 protein possesses over 80% identity with *M. tuberculosis *and *M. bovis *BCG proteins but serological and cellular responses to this antigen were highly specific to leprosy patients (or at-risk contacts). Antigen expression levels and bioavailability may determine the immune dominant antigens of each mycobacterial infection, such that differing responses to similar proteins may distinguish each disease. As a further demonstration of the limited ability of in silico predictions to indicate specificity, despite having no identified homologue in *M. tuberculosis*, the ML2346 protein induced strong IFNγ responses in all of the study groups, including TB patients. Based on these findings we suggest that, at present, in silico identification of *M. leprae *proteins with high identity to other mycobacterial proteins should not be used as a definitive criterion to exclude them from further diagnostic or vaccine evaluations.

Several studies have reported antigen-specific cellular and antibody responses during leprosy, but few studies have consolidated data to determine if particular antigens are differentially recognized across the disease spectrum [3,4,9,19-25]. Araoz *et al. *described *M. leprae *recombinant proteins recognized by antibodies produced by lepromatous patients that that could also induce CMI among tuberculoid patients [19]. Some of the proteins analyzed in our study (ML1632, ML1685 and ML1556) induced strong and specific production of IFNγ in TT/BT and HHC groups, demonstrating their potential application for identification of those at risk of developing tuberculoid leprosy. These proteins were not, however, recognized by antibodies from LL/BL patients. Our parallel screening suggested that the antibody response to *M. leprae *recombinant proteins was dependent upon their ability to induce cellular responses, and indicates only a limited number of *M. leprae *antigens contained T cell and B cell epitopes that are immune reactive in the context of disease (ML0405, ML2055 and ML2331). These antigens could be considered priority diagnostic antigens. In order to optimize the screening of new *M. leprae *antigens for leprosy diagnosis, our data suggest that it may be beneficial to conduct WBA testing before serological assays. If the protein is not able to induce a T cell response it could be de-prioritized from further testing.

## Conclusions

In summary, our results identify new *M. leprae *antigens that are recognized by antibody responses of lepromatous patients and cellular responses of tuberculoid leprosy patients. The identification of IgG-reactive antigens indicates the possibility of developing an improved serological diagnostic test for leprosy, especially if these antigens can be incorporated to supplement the current PGL-I based tests [10,11]. For the diagnosis of tuberculoid leprosy, our data indicate the WBA is a robust, relatively simple and user friendly format with which to screen and identify new diagnostic antigens. Together, these test formats would be desirable for field use in leprosy endemic countries and could contribute to the active detection of leprosy cases before the development of deformities and disabilities.

## List of abbreviations

BL: borderline lepromatous leprosy; BT: borderline tuberculoid leprosy; EC: endemic control; HHC: healthy household contact; IQR: interquartile range; LL: lepromatous leprosy; MB: multibacillary leprosy; MDT: multi-drug therapy; PB: paucibacillary leprosy; TB: tuberculosis; TT: tuberculoid leprosy; PGL-I: *M. leprae *cell wall phenolic glycolipid I; TCR: T cell receptor.

## Competing interests

The authors declare that they have no competing interests.

## Authors' contributions

LHS recruited patients, performed the experiments, analyzed the data and wrote the paper. MMAS conceived and designed the experiments, coordinated the field work, performed the experiments, analyzed the data, contributed reagents/materials/analysis tools and wrote the paper. RMO performed the experiments. ALMS recruited patients and performed the experiments. GCI performed the experiments and contributed reagents/materials/analysis tools. SGR performed the experiments, analyzed the data and contributed reagents/materials/analysis tools. MSD performed the experiments, analyzed the data, contributed reagents/materials/analysis tools and wrote the paper. All authors read and approved the final manuscript.

## Pre-publication history

The pre-publication history for this paper can be accessed here:

http://www.biomedcentral.com/1471-2334/11/26/prepub

## Supplementary Material

Additional file 1 **IFNγ production in WBA upon stimulation with PHA, MLCwA and PBS**. This figure indicates IFNγ production upon stimulation with positive controls (PHA and MLCwA) and the baseline concentration without any stimulant (PBS alone) used as negative control. Click here for file

Additional file 2 **IFNγ produced in WBA and OD of ELISA tests to detect IgG to Immunogenic *M. leprae *recombinant proteins**. The medium values of IFNγ and IgG ELISA optical density (OD) are shown in all study groups. Click here for file

Additional file 3 **Percentage of amino acid identity of immunogenic *M. leprae *proteins with proteins from other relevant mycobacteria species**. Blast Search for amino acid identity was performed using BLAST Uniprot (http://www.uniprot.org/). (-) = no homologue found. Locus_tag orthologous genes are indicated within parentheses. Click here for file
