# Supplementary figures and images for: Immunologically reactive M. leprae antigens with relevance to diagnosis and vaccine development
Source: BMC Infect Dis. 2011 Jan 26;11:26. doi: 10.1186/1471-2334-11-26 (PMC3040138; doi:10.1186/1471-2334-11-26)

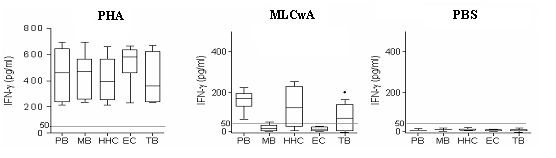

Supplement: Additional file 1 — IFNγ production in WBA upon stimulation with PHA, MLCwA and PBS. This figure indicates IFNγ production upon stimulation with positive controls (PHA and MLCwA) and the baseline concentration without any stimulant (PBS alone) used as negative control. [file 1471-2334-11-26-S1.TIFF]

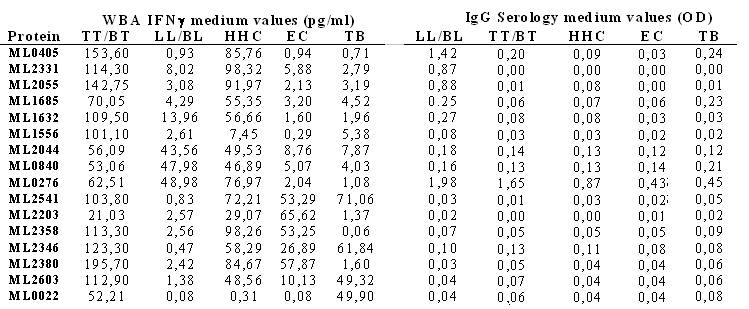

Supplement: Additional file 2 — IFNγ produced in WBA and OD of ELISA tests to detect IgG to Immunogenic M. leprae recombinant proteins. The medium values of IFNγ and IgG ELISA optical density (OD) are shown in all study groups. [file 1471-2334-11-26-S2.TIFF]

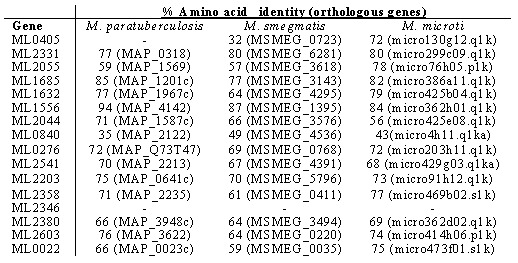

Supplement: Additional file 3 — Percentage of amino acid identity of immunogenic M. leprae proteins with proteins from other relevant mycobacteria species. Blast Search for amino acid identity was performed using BLAST Uniprot (http://www.uniprot.org/). (-) = no homologue found. Locus_tag orthologous genes are indicated within parentheses. [file 1471-2334-11-26-S3.TIFF]
